# On being and having: a qualitative study of self-perceptions in bipolar disorder

**DOI:** 10.3389/fpsyt.2024.1509979

**Published:** 2025-01-21

**Authors:** Matthew N. Ponticiello, Alexis L. Chang, Rebecca J. Chang, Salahudeen Mirza, Andrés Martin

**Affiliations:** Yale Child Study Center, Yale School of Medicine, New Haven, CT, United States

**Keywords:** qualitative, bipolar, meaning-making, mental illness, self-perceptions

## Abstract

**Background:**

Bipolar disorder (BD) is a chronic and often severe mental illness. Yet despite the well-documented complexities in its diagnosis and treatment, little research has been dedicated to understanding the complex inner landscape experienced by those living with BD. Even as qualitative research has explored the lived experience of BD across a variety of perspectives, i.e., what BD *looks like*, there is a lack of research exploring what BD *means* to those living with the condition. We conducted individual, semi-structured interviews with 20 adults with clinically stable BD to explore their perceptions of the condition, their construction of meaning of their illness, and their view of BD in relation to their sense of self. We coded the transcripts according to the principles of thematic analysis and analyzed the data using an interpretative phenomenological analysis approach.

**Results:**

We identified three overarching domains: (1) *Benefit or burden:* a dialectic through which participants weighed the valence of their illness over time; (2) *Self or other:* the internal or external locus through which they experienced BD; and (3) *From ineffability to meaning making:* the process of naming, understanding, and incorporating BD into their life’s whole. Within each domain, themes and subthemes outline nuanced and often conflicting perspectives of participants’ illness experiences.

**Conclusions:**

Across the varied and nuanced perspectives uncovered, our work provides a framework of three domains central to the inner reality of lived bipolar experience. Thoughtful understanding of patients’ experiences, perspectives, and desires within these three domains may aid clinicians and loved ones alike in more sensitively and effectively addressing the unique individual needs of those living with BD. It may also be informative for individuals living with BD themselves. By exploring patients’ perspectives in each of the three domains we identified, those with or at risk for BD as well as those caring for people with BD may be better positioned to help identify the inner work and practical interventions (such as finding bipolar community, or pathways to occupational thriving) needed to achieve a rich, meaningful life with BD.

## 1 Introduction

Bipolar disorder (BD) is a complex and chronic mental health condition characterized by significant mood fluctuations that include episodes of mania or hypomania and depression. The DSM-5 defines specific criteria for the diagnosis of bipolar subtypes, but the more recent concept of the bipolar spectrum has expanded the understanding of BD beyond the traditionally defined categories of bipolar I and II, and encompasses a range of mood disorders characterized by a spectrum of severity and varying degrees of mood elevation and depression (1).

Although studies conflict regarding the prevalence of BD, most estimates suggest a global lifetime prevalence between 1-2% (2–4). A large cross-sectional study across 11 countries found a lifetime prevalence of 2.4% for bipolar spectrum, including 0.6% for BD-I and 0.4% for BD-II (5). BD represents a significant burden as one of the leading causes of disability worldwide (6) and is associated with premature mortality, both by suicide and through co-occurring medical conditions (7).

Previous research, both quantitative and qualitative, has investigated the lived experiences of people on the bipolar spectrum. Quantitative quality of life assessment studies have consistently demonstrated lower functioning and well-being among people with BD, even in those considered clinically euthymic (8, 9). Qualitative research has aimed to better understand the subjective experiences of people living with BD beyond the predetermined outcomes typically used in clinical trials and other quantitative BD research. A significant portion of the qualitative literature has explored the impact on those living with BD, including difficulty with occupational functioning (10), impacts on relationships (11), feeling out of control (12), and self-doubt (13). These studies have largely emphasized the negative impacts of BD. By contrast, and despite a growing body of autobiographies and other popular literature taking a more positive tone toward BD (such as Kay Redfield Jamison’s *Touched with Fire*) (14), there have been few studies exploring positive aspects of BD, though some research has aimed to highlight what people living with BD view as benefits of the condition (15). Other qualitative research has focused on the experiences of those caring for people with BD (16), factors affecting medication adherence and beliefs surrounding the use of psychiatric medication (17), and challenges managing the condition and navigating healthcare systems (18–20).

A notable body of both quantitative and qualitative research has focused on the stigma surrounding BD, both public and internalized. BD is a highly stigmatized condition (21), and it has been well-established that this stigma leads to negative outcomes, including poorer psychosocial and occupational functioning (22), Conversely, reducing stigma can improve functioning, self-esteem, and engagement with healthcare (22, 23). Moreover, the sharing of personal narrative and lived experience can help reduce stigma (24, 25), highlighting the particular importance of qualitative research as a method of exploring the lived experiences of those on the bipolar spectrum.

There is a limited body of research exploring identity in BD. Inder et al. (13) performed qualitative thematic analysis of recorded psychotherapy sessions from 18 adolescent and young adult participants in a larger randomized controlled trial of therapy interventions for BD. They identified a theme of “problems in the development of a sense of self”, describing how changing moods and lack of stability created difficulty in developing a solid self-concept. Carls-Diamante (26) developed a philosophical taxonomy theoretically exploring ways BD might be incorporated into one’s self-concept, yet there remains a lack of research directly investigating how people on the bipolar spectrum perceive themselves in relation to their condition, beyond assessing challenges related to developmental identity formation.

Despite this rich body of work describing what life with BD *looks like* from the perspective of those living with it, there is a relative lack of research asking the question of what life with bipolar *means* to them. How do people living with BD think about the condition, how do they position their sense of self in relation to their diagnosis, and how do they shape meaningful lives living with BD? To address this gap in the literature, we conducted a qualitative study of individuals with clinically stable BD. Our specific focus was the participants’ construction of meaning of their illness, and their view of BD in relation to their sense of self.

## 2 Methods

### 2.1 Sampling and recruitment

We used snowball sampling (27), in which participants were asked if they knew anyone who was also on the bipolar spectrum and may be open to participate and referred them to the research team. The first participants were identified by the investigators and later advertisement via social media. In this way, we identified 20 participants (12 females) who were 18 years or older (median age 34, range 21 – 57); had a self-reported diagnosis of bipolar disorder (I, II, or mixed); and were clinically stable, defined by held consistent employment or enrollment in school, had been stably housed, and had not been hospitalized for the previous 6 or more months. The same definition of stability was used for all BD sub-types. Participants were pre-screened to determine whether they met this criterion. We provide further details about the study’s participants in **Supplementary File 1**. We continued recruitment until a wide range of perspectives were elicited via interviews, and continued until we reached thematic saturation. Saturation was further supported by consultation of the empirical literature suggesting that the final sample size was within the range typically required for saturation (9-17 interviews), especially considering the relative homogeneity of the final sample in terms of bipolar disorder subtype (75% BD-II) and clinical stability (28).

### 2.2 Data collection and transcription

Between February and July 2024, participants were invited to participate in a single, hour-long, in-depth interview. All interviews were conducted in English. We conducted individual, semi-structured interviews guided by an in-house guide organized into five key domains (sensitizing questions, perceptions of bipolar from others, ineffable mood states, romanticization/mania, and depressive phases). The semi-structured interview guide expanded over time and continued to explore self-perceptions of bipolar disorder and to ensure consistency across interviews (**Supplementary File 2**). Semi-structured interviewing is a flexible process that allows iterative revision of questions and is commonly used in qualitative research in healthcare to guide researchers to “co-create meaning” with participants through the elicitation of those feelings and opinions with a particular focus on potentially sensitive or personal topics. Participants are not necessarily asked all questions on the interview guide (29, 30). We recorded all interviews digitally over Zoom and transcribed them for analysis using Deepgram.

### 2.3 Reflexivity and positionality

As a study team including two individuals with bipolar II diagnoses and careers in medicine and academia (31, 32), we were additionally interested in the experiences of those living with bipolar II, and of “high-functioning” individuals on the bipolar spectrum (defined as those with stable work and/or study and housing conditions). These individuals were sought out as we hoped to capture the perspective of an under-studied group that is often poorly represented in studies of bipolar. Authors were keenly aware of their identities as people with BD while conducting this study. The authors were also mixed gender (three identified as cis-male and two identified as cis-female). Authors also came from a range of cultural/ethnic backgrounds including American, Mexican-American, Asian-American, and South Asian-American that promoted a greater understanding of the different cultural nuances surrounding mental illness in different cultures. All of these identities were reflected upon in relation to data interpretation to ensure integrity of findings.

We acknowledged positionality throughout the interviews, which drew greater attention to reflexivity during the analysis and manuscript writing (33). The two authors with BD diagnoses also took part as study participants; their duality of researcher-as-participant led to a deeper understanding of the content and enhanced triangulation, thus garnering further insights (34). This approach also parallels the “patient-as-partner” model of healthcare, education and research, where participants are welcome to provide feedback on results and contribute from the conceptualization of the project through the production of its manuscript (35). Lastly, our team drew from elements of Participatory Action Research, as investigators had simultaneous roles as participants, stakeholders, and beneficiaries (36).

### 2.4 Data analysis

We coded transcripts following the principles of thematic analysis (37). Thematic analysis emphasizes the active identification and construction of patterns that produce meaning across a given data set. Thematic analysis is a flexible and atheoretical exploration of rich qualitative data that allows researchers to construct themes from the data without necessarily developing a final theory. Our analysis, interpretation, and conceptualization were informed by an interpretative phenomenological analysis (IPA) framework (38–40). IPA is based in psychology and emphasizes the importance of acknowledging participants’ inner realities when identifying themes and constructing meaning from qualitative data. We followed an inductive approach in which research questions evolved beyond sensitizing questions to explore new and emerging themes.

We coded transcripts independently throughout the study in an interactive and iterative approach. We conducted interviewing collaboratively to allow for the identification of novel themes to inform subsequent interviews. Each transcript was reviewed by three authors. Coding began with an open coding approach followed by axial and then selective coding to further refine themes and sub-themes, relatively. Codes were entered into a coding matrix to be discussed in meetings with all authors. Authors then combined and triangulated codes to establish a final codebook, reduce redundancies, and refine domains, themes and subthemes. Any discrepancies were resolved through discussion and consensus. Final codes were supported by verbatim quotes from one or more participants. All interviews were analyzed using the final codes.

### 2.5 Ethics approval

The Yale School of Medicine’s Human Investigation Committee determined the study to be exempt of full Institutional Board review under 45CFR46.104 (2) (ii; Exempted protocol #2000036961, dated 01/13/24). All participants provided oral audiotaped consent, and were thanked for their participation with a $30 electronic gift card. In writing our findings, we adhered to best practices in qualitative research, as articulated in the COREQ guidelines (41).

## 3 Results

Through iterative thematic analysis we identified three overarching domains: (1) *Benefit or burden:* a dialectic through which participants weighed the valence of their illness over time; (2) *Self or other*: the internal or external locus through which they experienced their illness; and (3) *From ineffability to meaning making:* the process of naming, understanding, and incorporating the illness into a life’s whole. In **Table 1** we provide a summary and definition of these main domains and their underlying themes. We go on to describe each domain in the three subsections and corresponding tables that follow. We organized the tables following a similar rubric: (a) definition of each domain; (b) division into underlying themes and subthemes; and (c) support of constructs through representative quotations.

**Table 1:** Summary of domains and themes: self-perceptions among individuals with bipolar disorder (BD).

Self-perception	Definition
**Benefit or burden**	The degree to which BD is viewed as a net positive or a net negative on an individual’s life.
Enduring or “true” benefits	Aspects of BD associated with an adaptive advantage, e.g., increased creativity.
Temporary or “false” benefits	Those supposed advantages that may seem true at the moment but leave a negative path in their wake, e.g., decreased need for sleep.
Burdens	Aspects of the illness that have a clearly negative effect, e.g., suicidality.
**Self or other**	The way in which BD is lived as either an internal experience or through its effects on surrounding individuals.
Identity	How BD interacts with one’s identity, e.g., is it viewed as integral to the self, or as an added characteristic?
Intersectionality	How one’s identities interact with BD, e.g., how race, ethnicity, age, sexual orientation, or profession (among others) are relevant or not to the illness experience.
Others	How the people around impact one’s experience of BD, e.g., how acceptance or rejection by others can change someone’s experience.
**From ineffability to meaning making**	The process of moving toward an ability to name and describe the internal feeling states of BD.
Naming one’s experience	BD can be insidious and hard to identify, let alone articulate. This is a feature that can generally improve over time, going from an unknown to a better known state of mind.
Meaning of medication	Reconciliation to the role of medication and treatment can be crucial for long-term stability, and not infrequently start with denial, e.g., “I am not ill, I don’t need medicine.”
Towards a meaningful life	Incorporating BD as a reality and as a central part of the self is an important piece to living a full life in which the illness is acknowledged and embraced rather than ignored or dismissed. A strong therapeutic alliance can strengthen this and all other self-perceptions.

### 3.1 Benefit or burden

Given that the topic under discussion is a chronic, lifelong, and at times debilitating condition, it was notable how pervasive were *positive* views of BD by those living with it. Several participants used words like “benefit,” “gift,” or “blessing” as signifiers of how some positive aspects of their lives were enriched or amplified by their condition. Still, not all benefits were equal: some were “true” and enduring, while others were “false” – tantalizing and transient. Despite their description of positive aspects, most participants did not shy from describing the sometimes mild, sometimes devastating, burdens of living with BD (**Table 2**).

**Table 2:** Benefit or burden.

Theme	Subtheme	Representative quote
1.1. Enduring / “true” benefits	1.1.1. *Intra*personal: Psychological mindedness; insight; self-discovery; gratitude; resilience	One of the positives that has come out for me is just the ability to experience hurt and pain. It's something that I've learned from. It's something that I've gained insight from. (08M)
	1.1.2. *Inter*personal: Empathy; sensitivity; vibrancy; boldness	I think for a lot of us who have bipolar, we can just sense when someone's suffering. We're very intuitive, and we can just feel when somebody is suffering the way we have, and be able to really support them. And that’s a huge gift that we can give to the people in our lives, to the world. (19F)
	1.1.3. Creativity	I don't know why bipolar people have tended to be on the creative side. I don't know which is the chicken and which is the egg. (13F)
	1.1.4. Benefits *can be* worth the burdens	Interviewer: If I could wave a magic wand that would get rid of your bipolar today, would you take me up on that? Respondent: I don't think so. No, I don't think so. I think it's made me who I am today. (03F)
1.2. Temporary / “false” benefits	1.2.1. Increased outgoingness, output, productivity, drive	It just flowed: I wasn't writing so much as taking dictation. (05M)
	1.2.2. Romanticization	I've been told by many people, “I wish I would live this [hypomania], you know, just once.” But they haven’t fully calculated the negative effects that narrative can have, you know, the stigma, the negative perceptions, the pain of the illness. (16M)
	1.2.3. Benefits are *not* worth the burdens	I know some people don't wanna treat their mania, because they like the feeling. But for me, it's just like, yeah, it's a good drug, but it has a really bad comedown, no matter what. (10F)
1.3. Burdens	1.3.1. Misunderstanding and stigma	Mental health becomes weaponized. Because you might be saying something that’s true, pointing out a real injustice, but it's easier for the perpetrator to say, you know, it's false and that it's just a symptom of your mental illness. (04M)
	1.3.2. Shame, hypervigilance, sense of loss	We talk about recovery, but I don't believe in that concept. I have a professional life. I write. I go to conferences. I give papers. So I have a life. But I can't ever *recover* what I lost, those years that I lost. I have a hard time with the concept of recovery. So maybe that's why I'm thinking of it as hell. (17F)

#### 3.1.1 Enduring/”true” benefits

##### 3.1.1.1 Intrapersonal benefits

Participants described—and embraced—a number of personal characteristics that they felt their experiences with BD had bestowed upon them: psychological mindedness, insight, self-discovery, gratitude, resiliency.

I feel really grateful that I feel so deeply. I feel really grateful that I enjoy the entire spectrum of emotion. (ID# 01M)

Some participants felt that learning to navigate the complex internal experience of BD had honed their skills of introspection and insight:

It’s definitely made me think deeper into myself, and to analyze more deeply, whether it’s with myself or those around me. (ID# 03F)

The appreciation—or reappreciation—of joy and beauty was a common theme. For some, it related to a heightening of the senses, for others, to a lifting of the pain and darkness of an extreme mood state:

Before I got sick, I thought I knew what beauty was. I thought I knew what love was. I think that experience of suffering in that particular way, your mind changes your whole sense of who you are. You come out of that as so thankful, and that’s a spiritual state. You come out of that, and you can see beauty. (ID# 17F)

With few exceptions, participants reported a sense of strength and personal growth in emerging from episodes of the illness. Metaphors of “battles” and “fighting” were often used as participants described the resilience they’d developed:

I hear resilience, empathy, creativity, sensitivity. And definitely determination. Because you do have to keep fighting it. This thing can be relentless. (ID# 15F)

##### 3.1.1.2 Interpersonal benefits

Several participants described how their illness experience had strengthened old relationships or contributed to making new ones. Their experiences of suffering had deepened their empathy for the suffering of others, and their experiences of broad swaths of human emotion had sharpened their emotional attunement and sensitivity for others:

My therapist says that I have the capacity to connect with a fire hydrant. And I think that my capacity to connect with people comes from my ability to experience the entire spectrum of emotion. That kind of emotional attunement only comes from someone who’s experienced the whole gambit of emotion every single day. (ID# 01M)

In several instances, interpersonal relationships were enhanced through vibrancy:

As he puts it, I add color in his life, to his world. With bipolar, you have the affective intensity, a nice clinical term. Basically, you see things in brighter shades. (ID# 15F)

For others, increased interpersonal boldness during periods of mania or hypomania, while often causing strife, sometimes had lasting benefits:

I think that was challenging for my family to deal with, but I think we all kind of grew as a result from that because when I was hypomanic, I was able to communicate my needs more directly rather than just being like, oh, yeah, everything’s fine. (ID# 07F)

##### 3.1.1.3 Creativity

Many of our participants asserted a connection between BD and creativity, whether experienced through their own creativity or noted as they learned about their illness from others. Participants often pondered the source of this creativity. One suggested it had developed as a survival tool for finding alternative ways to manage her BD. Another suggested that her creativity originated as an outlet to express the chaos in her mind.

Creativity was described as related to mood states. Most felt their creativity flowed best during periods of (hypo)mania, but certainly not always:

I feel the most creative when I’m at either extreme of the spectrum. If I’m having a severe depressive episode, I find that my words come out the best in terms of writing. I am best able to capture my feelings and thoughts when I’m the most depressed. (ID# 14NB)

While participants generally noted the connection between BD and creativity with pride, one felt that BD was being given too much credit for this positive trait:

Bipolar doesn’t get credit for my creativity. (ID# 12F)

##### 3.1.1.4 Benefits *can be* worth the burdens

BD impacted all participants in ways small and large, from alienating a friend to near-fatal suicide attempts. Despite this wide range of outcomes, there was relatively little by ways of anger directed at the illness. We asked each participant to reflect on a thought experiment: if we could take their BD away with the wave of a magic wand, would they take us up on the offer? We found respondents evenly split. For the half who would keep their BD, the loss of the benefits described above, which in many cases had come to feel central to their identities, would be unacceptable:

I do love it, and I wouldn’t want to take it away because I think if you were to take the BD away from me, you would take part of my compassion. You would take part of my emotions. (ID# 13F)

For some, these benefits were considered as divinely ordained:

Regarding the magic wand, I think the reason I really wouldn’t think about is because of my faith that I believe God does everything for a reason, and he knows what’s going on. And this is the best thing for me, so I would definitely say no. (ID# 08M)

More common was a sense of magnanimous acceptance and ongoing working through:

I do perceive it as a gift. I’ve kind of trained myself and choose to see it that way. (ID# 19F)

#### 3.1.2 Temporary/false benefits

##### 3.1.2.1 Increased outgoingness, output, productivity, drive

Several participants highlighted perceived benefits of BD, almost invariably experienced during hypomanic episodes, that they acknowledged feel good in the moment but are fleeting, unsustainable, and may ultimately cause harm. From increased productivity and reduced need for sleep to the social lubricant of increased outgoingness and disinhibition, these temporary or false benefits were described almost longingly by some:

Hypomania is great. I wish that everybody could experience it, one day or another. You’re more productive. You have better ideas. People find you cooler. They are attracted to you, you know, you’re just magnetic. And so, every day I would wake up, and from the first minute to the last, it was just the best time I had. It’s just that it ends with depression, so it’s not sustainable. (ID# 16M)

For many participants, crashing from these periods of enhanced productivity and outgoingness had been devastating, driving doubt about what accomplishments might be “really theirs” versus symptoms of BD.

##### 3.1.2.2 Romanticization

A few participants, as described above, recalled the hypomanic experience as if seduced by its memory, and as if wanting to bring others into the fold. They acknowledged these descriptions as romanticizing:

I totally own the fact that when I talk about hypomania, it’s seducing. (ID# 16M)

Others noted prominent tropes that have generated a cultural narrative that romanticizes BD – the tortured artistic genius, the passionate but volatile love affair, etc. This romanticization was described, and criticized, by several participants as inaccurate and potentially damaging:

It may appear that hypomania is fun, productive, creative. But mania isn’t fun. Mania can kill you. Mania destroys your life, and it destroys the lives of those you love and even those you don’t know. So, it is over romanticized, and we have to be cautious. (ID# 04M)

Part of the cautionary tale is anticipating, and coming to terms with the fact that what goes up must come down, that

At the heart of every euphoria is a dysphoria. It’s just a matter of time. I think anybody who’s romanticizing bipolar disorder is probably pretty underinformed about the truth of the condition. (ID# 15F)

##### 3.1.2.3 Benefits are *not* worth the burdens

Among the half of participants who responded to our “magic wand” thought experiment by saying they would choose to be rid of their BD if given the option, the thought of full recovery, of not having to face the potential return of debilitating episodes, made the proposition an instantly appealing one:

Please tell me you’ve got that magic wand? (ID# 09F)

The benefits they’d experienced, whether “true” or “false,” simply weren’t worth the burdens:

It was nice to write a lot and sleep a little, but screw that. Screw the papers and give me my health. (ID# 05M)

#### 3.1.3 Burdens

##### 3.1.3.1 Misunderstanding and stigma

All participants reported feeling misunderstood or unseen at some point during the course of their illness. Individuals surrounding them, including loved ones and work colleagues, were described as often having little knowledge about BD. Family, friends and colleagues often responded awkwardly when learning about participants’ diagnoses and tiptoed around questions they may have perceived to be intrusive rather than supportive, or bought into hurtful stereotypes about BD. Several participants had been confronted with, and often internalized, explanations for BD’s etiology that centered on their volition, their weakness, or their outright moral failure:

I’ve always known I was mentally ill, but my parents ignored it and wouldn’t allow me to seek help for it. I remember being, like, 16. I told my dad I wanted to kill myself. And he was like, “Only cowards kill themselves. You’re not depressed. I’ve given you a good life.” (ID# 02F)

A burden shared by many of our participants with relatively well managed bipolar II was feeling unseen – their “high functioning” status had resulted in the additional burden of convincing the people around them that they still needed help, that they were still struggling with their BD:

I was always able to hold it together just enough that my grades weren’t slipping, but I felt like my brain was not cooperating with me the way it normally does. So, it can be hard to explain to people that you need help or get them to really understand what’s going on with you when your output is still good, when you’re able to hold that together so it doesn’t look like things are suffering that much. (ID# 07F)

Most participants described struggling with both internalized stigma and the effects of public stigma around BD. One participant said he chooses not to name his condition explicitly in any setting, acknowledging this is likely due to his internalized stigma. Others shared their experiences of discrimination related to their BD – a concrete form of enacted stigma. For example, three participants reported being fired from their jobs as a result of a new diagnosis. Two of them had stabilized and were doing well at the time they shared their diagnosis, yet they were let go, as their “instability” implied a liability their employers could not take.

##### 3.1.3.2 Shame, hypervigilance, sense of loss

An underlying thread was the misunderstanding – whether directed at participants by others or internalized – of BD as an illness of the will or even a moral weakness. These and other well-meaning but ultimately misguided “explanatory accusations” can contribute to the sense of shame so common among those affected by the disorder. Several participants also spoke of working through deep shame they felt as a result of actions they had taken or people they had hurt during the course of their illness:

Every day, I’m reminded of the things that I did in my manic episodes and my depressive episodes that have really delayed me or held me back in terms of future goals and those milestones that people that are in my age group are way ahead of me. (ID# 09F)

Given BD’s episodic nature, several participants spoke of their concern over a next episode: of being perennially on alert, looking for signs of recurrence. They reported that no matter how stable they may have become through treatment, they or their families read into the subtlest signs as indicators of a return of their illness, a hypervigilance that often felt exhausting and joy-dampening:

And the downside is now because I am so completely open about my diagnosis, I feel like a lot of people who are close to me in my life will see the happy days, and they’re like, are you just happy, or are you running manic? And it feels a little insulting because I wonder, am I not allowed to “just” be happy? (ID# 13F)

Many participants, years into their diagnosis, mourned what had been lost along the way of their illness:

I lost the kind of motherhood that I would have wanted, to give to my children and to have for myself as a mother. I lost years of their childhood. I lost years of remembering things. Every mother has a certain degree of guilt, I suppose, but mine is fueled by this illness. (ID# 17F)

### 3.2 Self or other

One of the central questions that participants pondered was the relationship between BD and their selves – illness or not, is BD an inherent part of themselves, or an “other,” a separate entity superimposed on their personhood? Just as BD played varying roles in participants’ identities, their varying identities affected their experience of BD, as did many “others,” from loved ones to the media to their faith (**Table 3**).

**Table 3:** Self or other.

Theme	Subtheme	Representative quote
2.1 Identity: How BD interacts with one’s identity	2.1.1 BD in relation to the sense of self	I'm not gonna say it frustrates me when other people say that bipolar is not who they are because I don't wanna take anybody's identity away from them, but I also get frustrated when people tell me I shouldn't identify as bipolar because they can't take it away from me either. (11M)
		I would say that since hypomania is not that distorting of your baseline personality, I think my illness is just an extension of who I am. So, like, I'm very social in my life, so hypomania was just me being extra social. (16M)
		It’s important to find outlets to speak about your experience as a reminder that you are not *only* bipolar, and you were just being controlled by something that is hereditary and genetically found in you, but can definitely be treated. (09F)
	2.1.2. Being or having	I feel like it's both: I *am* and I *have* bipolar. (02F)
2.2. Intersectionality: How one’s multiple identities interact with bipolar disorder	2.2.1. Cultural norms	Culturally, you know, Asian cultures, they wanna deal with things privately. There's a kind of invalidation as far as mental illness goes, and so there isn't a lot of support provided or, you know, they don't go out to seek support. (12F)
	2.2.2. Discrimination and bias	Cambridge graduate, model? White, tall, slim? Who gives a shit if you got bipolar? Nobody gives a … no one's gonna use that against you. But if you're a Black person and you're poor and you've got bipolar, good luck. (04M)
2.3. Others: How the people around impact one’s experience of bipolar disorder	2.3.1. Concealing and revealing	Sometimes I'll say I have bipolar, and I think they don't believe me because a lot of the times I'm masking. I spent a lot of my energy masking, and I'm also just known to be a stoic person. (02F)
	2.3.2. Optimal and unhelpful responses	To this day, I can count with the fingers of half a hand how many people have asked how I'm doing. (05M)
	2.3.3. Family and generational impacts; children and reproduction	We're trying to start a family, and I decided years ago that I would not pass on my own genetic material, that we'd use an egg donor instead because I would not wish my experiences on my worst enemy, never mind my own child. (15F)
	2.3.4. Support, online community, and spirituality	I probably would feel a lot more crazy without bipolar Instagram. Like, I don't feel crazy now because of the community. And because I'm able to talk about it and express myself and say things that I know they're not gonna judge me. One person told me, I have gotten so much more from this group than I have anywhere else, like from doctors or medication. (12F)
	2.3.5. Media depictions	People will be like, what's a good movie to understand bipolar better? And I'm like, honestly, there's none. Because they're all right or wrong for any one person that's watching it. (13F)

#### 3.2.1 Identity: How BD interacts with one’s identity

##### 3.2.1.1 BD in relation to the sense of self

Participants had widely varying notions of where BD exists in relation to one’s identity and their inherent sense of self. For some, it was a completely separate entity – one that affected them significantly but was not a true part of them. One participant considered BD as a separate person:

A way I like to think of bipolar is like another person, one that’s deeply related to me. They’re always there, and they’re not a good person to keep around a lot of the time, like they’re pushing me to do things I shouldn’t or reminding me about things I shouldn’t focus on or whatever. So, it’s extremely distinct from me in that it’s another person, but it is a person that I have a very strong, important, entangled relationship with. (ID# 20M)

Others felt that BD was deeply intrinsic to who they were, in one case even denying the concept of BD as an illness:

I don’t separate bipolar disorder from me. Like, I think it *is* me. I don’t even consider bipolar an illness for me. (ID# 01M)

Participants who endorsed a biomedical view of BD, whether stated directly or indirectly, often perceived their BD as no different from any other medical condition – as a separate entity with no overlap with their intrinsic sense of self:

I definitely haven’t identified with it, meaning that it’s something I have. It’s something I need to take care of. It’s something I need to make sure I don’t let my guard down about. But just like any other type of illness that just needs to be treated properly. I don’t think I would say that it’s, like, me. It’s something I have to take care of. That’s it. (ID# 08M)

Many participants landed somewhere in between, acknowledging the sweeping effects of BD across their lives, which inevitably shaped their identity to some degree, while keeping some distance between their intrinsic sense of self and their illness:

For me, honestly, it’s just another thing. Like I say, I have red hair. I have bipolar disorder. It affects my everyday life and just about every decision that I make, but it’s not a part of who I am as a human. It’s just something that’s fundamental to my experiences. (ID# 15F)

Still, some participants described challenges in parsing out what specific parts of themselves were “really them” versus a symptom of BD:

It’s this conundrum. I know that I have this. I know what the symptoms are, but I still don’t know who I am. And I don’t know if I ever will, and I don’t know if when I’m behaving in a certain way, is that me? My overconfidence when I’m hypomanic, does that mean I’m not a confident person? (ID# 12F)

A recurring theme was that as time elapsed, the distance between one’s diagnosis and identity grew:

When I was diagnosed with it, it was most of my identity. 99% of it was bipolar, and 1% of it was me actually knowing who the hell I was. But over time, it’s taken up less and less of that overall spectrum of who I am. (ID# 11M)

##### 3.2.1.2 Being or having

We also explored the language participants preferred to use when describing their relationship to BD. In particular, choices of “I *am* bipolar” (akin to “person-first” language) vs “I *have* bipolar” (akin to “condition-first” language) came up repeatedly. Participants had varying preferences:

I don’t like saying “I am bipolar” because I’m more than bipolar. You know? It doesn’t define me. (ID# 18F)

And for me to say “I am bipolar” feels like I’m fully taking control of it and fully taking it as part of me, which feels more like, hey, I’m embracing it, which means I can manage it. (ID# 13F)

The language choice largely seemed to be an extension of the locus through which participants viewed their BD, as described in 3.2.1.1. Those who viewed bipolar as an intrinsic part of themselves often embraced “I am” language, while those who viewed their BD as a diagnosis separate from their sense of self often preferred “I have” language. Some participants also felt that “I have” language was a less stigmatizing way of speaking about BD.

That being said, multiple participants acknowledged the wide variety in preferences that exists among people with BD, with nobody claiming a consensus in the broader “bipolar community” over what language is most appropriate.

#### 3.2.2 Intersectionality: How one’s multiple identities interact with BD

##### 3.2.2.1 Cultural norms

Just as BD had a variety of relationships to participants’ identities, participants’ intersectional identities – race, gender, age, queerness, religion, socioeconomic status, and more – impacted their experience of BD. In particular, participants talked about how the cultural norms of their racial or ethnic communities of origin impacted everything from the attitudes of their families toward their BD to the mental health care they were allowed to access:

I can’t speak for all ethnicities, but I know in Mexican families like mine, to have a mental illness is seen as a weakness. It was very hard because it was seen as, like, I was just being lazy. (ID# 13F)

I feel like mental illnesses runs more rampant in the Black community and it’s not treated because our people don’t understand or, well, they don’t want to understand. You know, they’re very religious, and they think that they can pray anything away. (ID# 18F)

Many participants brought up similar examples of the particular attitudes of their racial or ethnic communities (from White Midwesterners to Black South Africans) adding a layer of challenge to life with BD. In contrast, some younger participants noted that younger generations tend to be more understanding and accepting of BD and other mental illnesses, as do members of the LGBTQ+ community:

I think at least in our generation, at least in the circles I socialize in, it’s, like, completely unstigmatized to have a bipolar diagnosis. It’s not normal, but it’s understandable. [ … ] A lot of people in my circle are queer, and I think queer people may just have a better understanding of certain things. (ID# 14NB)

##### 3.2.2.2 Discrimination and bias

Discrimination and bias related to BD were most often described *within* individuals’ communities. However, some described how discrimination presented a unique burden for those with intersectional marginalized identities. One participant summarized the unique burden of living with BD as a member of minoritized communities – and why the intersectionality of one or more marginalized identities with mental illness matters:

Maybe because of my ethnicity as well, maybe because I’m Muslim, the intersectionality, you know. Like, the definition of stigma is a deeply discrediting attribute. Right? That’s how Goffman defines stigma. And it’s like, well, how many deeply discrediting attributes are you allowed to have? You know, you’re Muslim. Okay. But you are also a person of color, and you also have a severe mental illness? You know, you’ve crossed the line there. (ID# 04M)

Gender was another identity that intersected with BD. One participant brought up the use of the word “bipolar” as a gendered derogative used to discredit women:

A lot of the men that I’ve dated have an idea about what bipolar disorder is or what a bipolar woman looks like. They’ve called exes “bipolar” inappropriately, or incorrectly. And so, then I’m like, “Oh, was she actually bipolar, or was she just mad at something you did and you just called her that to dismiss her?” (ID# 13F)

#### 3.2.3 Others: How the people around impact one’s experience of BD

##### 3.2.3.1 Concealing and revealing

The decision to share one’s BD diagnosis in social and professional settings was complex and personal for many, with the potential to have life-altering consequences. Participants described conflicting motivations – a desire to be open, honest, and even proud of their diagnosis; versus fears ranging from awkward responses to backlash and discrimination. Others expressed a desire to make it a “non-issue”:

I just wanna be open about it. I don’t want bipolar to be a “thing” for me. I just want it to be, like, what it is. Fine. I have a diagnosis. Wonderful. Great. Let’s move on. (ID# 08M)

Beyond the issue of telling others their diagnosis, multiple participants brought up the idea of masking – a term originally used in the autistic community to describe changing one’s behaviors in order to “pass” as neurotypical and avoid the stigma associated with autism. In the context of BD, participants particularly described masking during depressive phases by “putting on a happy face.” While masking provided some protection from social discomfort or stigmatization, participants also reported downsides such as dismissal or disbelief from others after sharing their diagnosis:

It’s just frustrating because my mom didn’t believe I had it, because she just views me as, you know, okay and fine. Because I do mask it quite a bit, especially if I’m depressed. I’m not, like, teary-eyed when I’m depressed or anything like that. Quite the contrary, I’m nothing. (ID# 10F)

##### 3.2.3.2 Optimal and unhelpful responses

When participants chose to share their diagnosis, the responses of the people around them had an enormous impact. Many shared stories of deep hurt that resulted from the early responses of friends and families, and some of these wounds felt raw even years later. Commonly cited unhelpful responses included awkwardness and discomfort talking about mental health, disbelief (e.g., “Seriously? I would have never guessed you were bipolar!”), minimizing (e.g., “at least you have a roof over your head”), drawing comparisons with other mental illnesses, and dismissal (e.g., “I don’t have to listen to you because you were (hypo)manic when you said that”). One participant described the emergence of his BD as a culling force in his relationships:

You know that famous quote, right? “Champagne for my real friends and real pain for my sham friends.” If you wanna know who your true friends are, have a manic episode. (ID# 04M)

Another participant acknowledged how difficult it can be for the loved ones of those living with BD to “get it right,” even when they have the best of intentions:

I think there’s often a disconnect between how much folks want to support us and how much they actually can; what capacity they have. To really be able to support someone with bipolar disorder, you have to be in a good spot yourself, to be willing to be with your own kind of shadows and darkness, especially to support folks in the depressive state. (ID# 19F)

Participants expressed varying preferences for how they hoped people would respond to hearing about their diagnosis. For example, one participant wished people would “play it cool” when he shared his diagnosis, while another perceived her family’s understated reaction as invalidating of the suffering she had experienced. However, one near universal desire arose: just ask. Ask how you can be supportive, ask what would be helpful, and come in with an open heart:

You don’t have to understand my experience. You just have to want to understand it. (ID# 12F)

##### 3.2.3.3 Family and generational impacts; children and reproduction

BD is highly heritable, and many participants had at least one family member with suspected or confirmed BD – including in older generations for whom severe mental illness often meant institutionalization or incarceration – in many cases leaving a family legacy that affected participants today:

Just to give you some background on my family, my grandmother was institutionalized. She had shock therapy. So my parents have a pretty skewed idea of mental health and therapy and all of that. (ID# 03F)

Just as participants shared about their families’ past relationships to mental illness, they also looked ahead to the future – namely, what BD meant for them in terms of family planning. Three participants said they had long ago decided they would never have biological children for fear of passing on the illness; one was currently in the process of using an egg donor and surrogate carrier (to avoid the risks associated with going off BD medications) to have a child. Another longed to have children, but had also ruled out the possibility of adopting, due to the unpredictable nature of BD:

I probably won’t be in a position to adopt kids either at any point. I would love to. I always wanted to. Do I think I’d be capable? Do I think I have support systems in place? Maybe. Maybe. But the problem is that answer is always going to be maybe. There’s no way of knowing that the answer is yes. (ID# 11M)

Still, many other participants already had biological children or were open to it, and while some were concerned about the possibility that their children could develop BD, they recognized that their own experiences equipped them well to catch it early and care for their children should that possibility occur:

Yeah, it’s a preoccupation. But I feel like if my child ever develops bipolar, I’ll be so attentive and, you know, so focused on his development, as I have had it myself, that I’m hopeful that he will get good treatment. (ID# 16M)

##### 3.2.3.4 Support, online community, and spirituality

Support (outside of psychiatric care and psychotherapy) for our participants came in many forms, including family and friends, in-person support groups, spirituality, and online community. In part due to our recruitment via social media, several of our participants were highly involved in online BD communities, following and in some cases running accounts on Instagram, TikTok, and/or YouTube with thousands of followers. For people living with BD, these accounts together form an online community of peer-to-peer information, support, advice, and, importantly, a sense of belonging:

I love the bipolar community on Instagram. I feel like we all follow each other. It’s like this weird sense of tribe, I guess, to know that I’m not alone. It feels really great to just be able to access that at any time, which is really helpful because I think years ago, people weren’t as open about it. (ID# 13F)

One participant even said she wished psychiatrists would “prescribe community” (ID# 12F) to people newly diagnosed with BD; such was the importance of the online bipolar community to many of our participants.

Spirituality and religion were another important source of support, but a double-edged sword: while many participants felt a sense of comfort, strength, and support from a higher power, many also had experienced stigma, misunderstanding (though sometimes well-intentioned), or abandonment from their religious or spiritual communities as a result of their BD. For one participant, manic episodes involving religiously themed delusions had created a strain between him and his religious community; another was part of a New Age spiritual community that disdained her for taking prescription psychiatric medication instead of psychedelics.

So I had, I had, you know, a person like that, like, last year just kind of, say something that enraged me where he was just like, yeah. You know, I used to have cyclothymia, and I cured it through psychedelics. I’m like, cool. I’m glad that worked for you. And he just kept going and just kept trying to convince me. I’m like, dude, I went off my medication last year, and I almost killed myself. (ID# 19F)

Unfortunately, not one of our participants described their religious community responding well to their BD.

##### 3.2.3.5 Media depictions

The popular media can be described as an “other” – a broadly defined entity that affects people living with BD. Films, TV shows, celebrities, and other publicly visible depictions of BD were a double-edged sword. While positive (or even simply accurate) depictions were appreciated as a source of visibility or as a resource for helping loved ones understand the bipolar experience, negative or inaccurate depictions could drive stigma and misunderstanding. For example, some participants were grateful for the visibility celebrities have brought to BD:

I think it’s great. I’m very grateful for the celebrities that have spoken out. I think it’s made it a lot easier for everyone to speak out about it because these are public figures that are overall really respected, and they’ve given, you know, a face to bipolar. (ID# 19F)

However, one name came up repeatedly, in a mostly negative light: Kanye West. The rapper is a polarizing figure, and is one of the world’s most visible people living with BD – among younger generations, his name is practically synonymous with BD. Though regarded by some as a creative genius, his erratic and often problematic public behavior has led some of our participants to feel he gives a bad name to BD:

You see people like Kanye, you know, he’s one of the big bipolar people that people know of, and he’s not the kind of person that a lot of us wanna be associated with. (ID# 07F)

Participants also acknowledged that no one depiction of BD could possibly feel accurate to every single person with BD, due to the heterogeneity of lived experiences with BD.

### 3.3 From ineffability to meaning making

Making meaning out of the often mystifying and distressing experience of BD arose as a central theme. Participants sought to understand and name their life’s journey with BD – from the time of diagnosis, when experiences that participants had long struggled to make sense of or even describe in words began to take clarity, to the ongoing process of shaping a meaningful life with BD (**Table 4**).

**Table 4:** From ineffability to meaning making.

Theme	Subtheme	Representative quote
3.1. Naming one’s experiences	3.1.1. Ineffability	I was relieved. Something "was up," but I didn't have words to explain what was up, I guess. And now I do. (14NB)
	3.1.2. After the diagnosis: Relief and fear	I was honestly a little thrilled and relieved. It felt to me like, okay: If I can name it, I can manage it. (13F)
		I'm like, oh, shit. I guess life is basically over. This is it. (11M)
	3.1.3. A new lens	Being recently diagnosed, I have been doing a lot of reexamining and reviewing my life through that lens, of rediscovering myself through the diagnosis. (12F)
3.2. Meaning of medication	3.2.1. Shame, ambivalence, and acceptance	I think I didn’t want to take medication because I wasn't fully convinced that I … I was still telling myself, you should be able to just control your emotions, and I wasn't fully convinced that it was something that, like, deserved medical treatment. (06F)
	3.2.2. A concession to biology	I kind of hate that medication is working for me right now, because it rather disproves my worldview of, like, medications are stupid; that we can solve most mental illness through therapy. (01M)
3.3. Towards a meaningful life	3.3.1. Vocation and advocacy	Our illnesses can actually help in our [pastoral] work. Insofar as they give us a certain degree of insight. Although you can't map your own experience onto somebody else, people know you're not going to judge them, and that's huge because this sort of thing, people judge, especially in the religious world. (17F)
	3.3.2. Thriving	I think I was interpreting “high functioning” on a much more superficial level of getting my work done. I was doing it really well, which is what I thought meant “high functioning.” But she was saying that being a well-balanced and well-adjusted adult is more than that, it’s flourishing interpersonally, beyond just getting your work done. (01M)

#### 3.3.1 Naming one’s experiences

##### 3.3.1.1 Ineffability

Many of our participants described the distress of BD as amplified when unable to be explained – that is, in its ineffability. While describing their experiences in our interviews, some found that there was something about BD that was simply beyond language:

There’s something terribly horrible about hypomania; I’m healthy now and it’s very, very difficult to convey in words what was so horrible. I mean, I could describe depression like Eskimos can describe snow, but I don’t quite know how to do justice to the terribleness of this thing. (ID# 05M)

For many, this ineffability was most distressing in the period between onset of symptoms and receiving a diagnosis – when they were experiencing symptoms of BD but didn’t have the words to name what was going on.

##### 3.3.1.2 After the diagnosis: Relief and fear

Participants often described receiving their diagnosis as a major turning point in their lives. For many, diagnosis brought a profound sense of relief – if not a reprieve from BD’s symptoms, at least from the distress of their inability to put the experiences into words, which often left participants feeling like they were “just crazy”:

It was like this euphoric feeling almost of, like, I get it. What has felt like I’m just crazy is now something that I can label and put a name to. And also, something that I can get help with. That it’s not something that’s just completely out there in the ether. (ID# 08M)

On the other hand, participants also described feeling fear or even despair after receiving their BD diagnosis:

To me, bipolar sounded not just life changing but life breaking, like it was an announcement that my life would suck for the rest of my days. (ID# 16M)

##### 3.3.1.3 A new lens

Regardless of their initial reaction to their diagnosis, many participants came to see it as a new lens through which to examine their life experiences. Being given a name for their condition allowed them to reframe and better understand their experiences, their behaviors, and their relationships – often emerging with a sense of clarity:

It allowed me to really look at my life in the past and in the present with a much clearer lens. I made more sense to myself, and I felt like I’d lacked self-awareness for a long time before then. But giving that kind of framework, the diagnosis has helped me to really see myself more clearly. (ID# 14NB)

This new lens, in many cases, also helped participants to work through the shame that years of living with undiagnosed BD often brought:

I feel like it’s slowly wearing off, wearing away my feeling shame about myself. I’m not saying that the shame and guilt is completely gone. But at least I’m able to put it next to me to examine instead of carrying it with me all the time. (ID# 12F)

#### 3.3.2 Meaning of medication

##### 3.3.2.1 Shame, ambivalence, and acceptance

Participants had mixed and complicated feelings about taking psychiatric medication to manage their BD. For some, despite acknowledging the importance of their medication or expressing gratitude for the ways medication had changed their lives, there lingered a nagging sense of shame:

I know medicines are so important, but it just feels wrong to take, like, 7 pills every day. I don’t know where that stigma comes from. Well, it feels like I need these pills to function, which I kinda do, so it just feels like I’m dependent on something. (ID# 02F)

Others felt ambivalent about medication, wishing they could manage their BD without it, or feeling unconvinced that their condition was even something that required medication in the first place.

I think, I also just, I wasn’t fully convinced that I … I was still putting myself in the realm of you’re not able to control your emotions, and then I wasn’t fully convinced that it was something that, like, deserved medical treatment. (ID# 06F)

For others, coming to terms with the likelihood of taking daily medication indefinitely was a key part of coming to accept their BD as a part of their lives:

I’m coming to a place where I’m fully at peace with the fact that I have bipolar disorder. I’m going to take medication for the rest of my life. I’m also a very spiritual person and believe in, you know, plant medicine, all these different things, but I need to take my bipolar medication because it allows me to stay alive and do my work in the world. (ID# 19F)

##### 3.3.2.2 A concession to biology

Among participants who had negative feelings toward psychiatric medication, the most common reasons were side effects, shame, and stigma. However, three participants presented a different perspective: That to take psychiatric medication would be a concession to the biomedical (and hegemonic) view of BD, that taking medication means chalking BD up to brain chemistry while ignoring factors like trauma in the causative pathway of BD:

I wasn’t too keen on taking medication. I thought it would be like a concession, that it’s all down to our brain chemistry. Right? It wasn’t because of the trauma. It wasn’t because of the racism. It wasn’t because of discrimination. It wasn’t because of the injustice. No. It was because of me and my brain chemistry gone awry. (ID# 04M)

#### 3.3.3 Towards a meaningful life

Beyond learning to manage BD symptoms and coming to terms with BD as a lifelong condition, many participants described the process of incorporating BD into a life that felt meaningful to them.

##### 3.3.3.1 Vocation and advocacy

For many of our participants, their lived experience with BD had significantly shaped the course of their careers, in some cases described as a “calling”. For some, BD had either generated or deepened a passion for a career as a mental health professional:

I feel like it’s deepened my passion for what I do as a social worker. As a social worker, it gives me a deeper understanding of people and how much people go through, and that life is not easy no matter where you come from and who you are. (ID# 03F)

Others felt that BD had guided and strengthened their skills and passion for vocations outside of medicine or mental health, such as clergy, that still draw heavily upon traits such as the empathy and open-mindedness they attributed to their BD. Multiple participants were either volunteers or held paid positions in mental health advocacy, sharing with us their passion for bringing awareness, resources, and equity in mental healthcare access for others:

It just amazes me just how many people need resources, and they don’t even know. They don’t know that any of this exists. (ID# 18F)

BD also shaped the careers of some participants in a more practical way. Recognizing their BD-related needs for routine, adequate sleep, and in some cases, creative freedom, these participants had built careers across a wide spectrum of industries that allowed them to set their own hours and maintain the flexibility they needed to manage their BD.

##### 3.3.3.2 Thriving

Many of our participants, having relatively well-managed BD and in stable employment or education (as per our inclusion criteria), felt that this stability was just the first step in creating a meaningful life with BD. These participants sought not just to survive life with BD, but to *thrive* – and, often, to help others with BD come to thrive as well:

And so getting diagnosed, figuring out the best treatment possibilities, all of those things are great, but the next step for me is, like, yes, we need to get people to the point of where they can be in public, but beyond that how can we get them to where they can see themselves with the option of thriving, of being an additive to the community and bringing value and creating that for themselves? (ID# 11M)

## 4 Discussion

Qualitative analysis of interviews with people with BD revealed three overarching domains that shape people with BD’s sense of self and relationship with their condition: the dialectical relationship between the good and bad that accompanied their condition (*benefit or burden)*; how BD interacted with one’s identity (*self or other*); and how individuals created meaning through their illness to integrate BD into their life’s whole (*from ineffability to meaning making)*.

Previous studies on BD have typically focused on either the benefits or burdens of BD. Similar burdens were found in our study such as occupational challenges (10), stigma and discrimination (22, 42), and feeling misunderstood (43). The similarity in our findings is likely due to the consistency of the destructive components of (hypo)mania and the debilitating aspects of depression. A unique burden found in our study was family planning and the ways BD created complex decision-making around genetics, pregnancy, and ability to parent. In addition, many of the benefits of BD (both temporary and enduring) found in our study, including enhanced productivity, strengthened affect, creativity, and better interpersonal connectedness, have been described elsewhere (15). It is also worth noting that people’s appreciation of their bipolar may come from a desire to resolve cognitive dissonance and reach acceptance, as opposed to actually enjoying their experience with BD. Thus, highlighting positive aspects of BD may serve as a form of adaptation to life with the condition.

Identifying how BD interacted with participants’ sense of self was a critical element of this study. There is a dearth of literature on identity and BD. One of the few papers published on the topic is Carls-Diamente’s “*Know thyself: bipolar disorder and self-concept*,” which presents a philosophical taxonomy of four different possible relationships between the self and BD: “BD contributes to the self,” “BD scaffolds the self,” “BD gradually becomes part of the self,” and “BD is not part of the ‘real self’” (26). Here, we contribute to this theoretical framework by presenting empirical data that can be situated within it. Within our participant sample, we achieved thematic saturation across varying perspectives that agree with most elements of this taxonomy to varying degrees, with the exception of “BD gradually becomes part of the self” – in contrast, we found that participants tended to accept their condition more, enabling them to develop greater separation between BD and their sense of self. It is also worth noting that while a minority of our participants aligned with Carls-Diamante’s fourth relationship, “BD is not part of the ‘real self,’” many of our participants strongly endorsed “being” bipolar, feeling that BD was an inextricable and inherent part of who they were. Our findings suggest that BD is a part of the ‘real self’ for many people living with it. Furthermore, other studies on BD and identity development describe BD as negatively contributing to identity formation, causing confusion, contradiction, and self-doubt (13). In contrast, we found that many participants derived a great sense of strength, resilience, and compassion from their BD that contributed to their identity formation. This may be because many people with BD face great adversity. The ability to create meaning in the face of adversity is a critical way to develop resilience and move towards self-actualization (44–46), both of which may aid in identity formation.

Another interesting finding in our study was the relationship of BD to occupational challenges. BD can be presented as something in conflict with consistent employment or professional success (47, 48). While some of our participants did struggle with employment, our study provided a richer view of how participants navigate the challenges of employment while living with a chronic mental illness. In some cases, participants’ commitment to and passion for their work was strengthened because of their relationship with BD. Other participants decided to make career switches and lean into the mental health space for work and volunteerism. Here, we see how people with BD can not only just “survive” in the workforce, but actually “make it” and lead rich professional lives.

The power of having a community of people with BD was a particularly salient theme among people with BD. While some participants’ cultural backgrounds stigmatized or invalidated their mental illness, numerous participants used social media to connect with other people with BD, describing the power of finding a community of people with shared living experience. Those who had started BD-oriented social media accounts that blossomed into significant followings felt a real sense of pride and fulfillment in having created this transformative community. Some made friends through these online communities, while others simply enjoyed relatable memes and funny videos made by people with BD. Some of our participants were found via social media and via snowball sampling across friendships developed in online BD communities. These findings suggest that people with BD may benefit from organizing and participating in these informal support groups online (49). This also speaks to the importance of “prescribing community” among clinicians and approaching BD through a biopsychosocial model.

Finally, through its Participatory Action Research framework, this study aimed to produce knowledge about BD from the BD community. By doing so, we begin to move forward in understanding (50) the bipolar community versus explaining BD psychopathology, thus humanizing the condition and those living with it. By sharing personal narratives and lived experience with BD, like in this study, we can also move the needle forward in destigmatizing BD – a key component to identifying and treating mental illness (51, 52). Furthermore, this paper is well-positioned to help various parties (friends and family members of someone with BD, clinicians, the general public) understand and treat BD. Particularly as it pertains to treatment, addressing internalized stigma may be a key component of BD treatment as well as linking patients to support groups and other forms of community.

Our study has some limitations. First, snowball sampling often means participants will suggest other participants within their social networks. This could lead to homogeneity of participants. This may particularly influence our findings as a handful of participants belonged to the same or similar communities on social media. Also worth noting is that all of our participants were at least partially college-educated, suggesting a certain homogeneity in socioeconomic status. Further, self-selection bias may have occurred as a result of purposive sampling. Those who agree to participate may have stronger thoughts or feelings about BD. In addition, given BD is heavily stigmatized, some individuals may have not felt comfortable coming forward to participate in our study. A handful of people we approached denied their diagnosis or declined participation, in some cases without an explanation. Another consideration is that we only included people with a self-reported diagnosis of bipolar disorder, and who were relatively stable. Consequently, we may have missed certain sub-populations with different perspectives on BD given their experience of symptoms without formal diagnosis or with unmanaged symptoms. Qualitative data is also highly contextual and not meant to be generalizable. Despite the limitations of this study, this study had numerous strengths. First, the study population was unique in that it included people with BD who were particularly high functioning, highlighting perspectives on BD that are often overlooked. Second, all participants were given the opportunity to provide feedback, strengthening the validity and reliability of our findings. Third, we included individuals with BD I and II, addressing the relative paucity of research on individuals with BD II (53). Lastly, qualitative research is particularly well-suited for answering our research questions as it is highly specific, considering the nuances of each participants’ lived experience.

## 5 Conclusion

In this paper, we elucidate self-perceptions of people with BD, empowering them to create their own narrative regarding their experiences with their illness. Many of our participants describe a nuanced experience of BD that is not defined by either the highs of (hypo)mania or lows of depression. Across a wide spectrum of perspectives on the relationship between BD and the self, many described empowering means of integrating BD into their way of being and living. This suggests a spectrum of ways that people with BD can live full, meaningful lives. This paper may serve as a resource for clinicians, loved ones of those with BD, and the general public by presenting a framework of three domains underpinning the inner living experience of BD. By thoughtfully exploring patients’ perspectives in each of these domains, those caring for people with BD – as well as those with BD or a liability to BD themselves - may be better positioned to help identify the inner work and practical interventions (such as finding bipolar community, or pathways to occupational thriving) needed to achieve a rich, meaningful life with BD.

## Data Availability

The raw data supporting the conclusions of this article will be made available by the authors, without undue reservation.
