# Supplementary material for: On being and having: a qualitative study of self-perceptions in bipolar disorder
Source: Front Psychiatry. 2025 Jan 21;15:1509979. doi: 10.3389/fpsyt.2024.1509979 (PMC11790665; doi:10.3389/fpsyt.2024.1509979)
Supplement: Supplementary file 1 [file SupplementaryFile1.docx]

Supplementary Material

# Participant Demographics

| **Characteristic** | **Summary** |
| --- | --- |
| **Age, Median (IQR)** | 37 (25–45) |
| **Race, n (%)** |  |
| White | 12 (60) |
| Asian | 2 (10) |
| Mixed (Asian, White Latina) | 4 (20) |
| Black | 2 (10) |
| **Gender, n (%)** |  |
| 12 female | 60% |
| 7 male | 35% |
| 1 non-binary | 5% |
| **Educational Attainment, n (%)** |  |
| Doctorate | 3 (15) |
| Graduate degree | 4 (20) |
| Some graduate school | 4 (20) |
| Bachelor's | 8 (40) |
| Some bachelor's | 2 (10) |
| **Relationship Status, n (%)** |  |
| Single | 12 (60) |
| Married | 6 (30) |
| Divorced | 2 (10) |
| **Diagnosis, n (%)** |  |
| BD-I | 5 (25) |
| BD-II | 15 (75) |
| **Age at Diagnosis, Median (IQR)** | 23 (20-36) |
| **Ever inpatient care for condition, n (%)** | 9 (45%) |
| **Lifetime experience of suicidality, n (%)** | 18 (90%) |
| **Lifetime psychotropic use, (%)**  Lithium | 9 (45) |
| Valproate | 2 (10) |
| Lamotrigine | 16 (80) |
| Antipsychotics | 15 (75) |
| Benzodiazepines | 11 (55) |
| Antidepressants | 17 (85) |
