# Supplementary material for: On being and having: a qualitative study of self-perceptions in bipolar disorder
Source: Front Psychiatry. 2025 Jan 21;15:1509979. doi: 10.3389/fpsyt.2024.1509979 (PMC11790665; doi:10.3389/fpsyt.2024.1509979)
Supplement: Supplementary file 2 [file SupplementaryFile2.docx]

Supplementary Material

**Supplementary 2. Interview Guide**

1. As you have seen from the shared document, I have a broad range of questions for you, but before we go there, I want to ask if there are things you’d particularly like us to touch upon (or not)?
2. **Self-perceptions of bipolar disorder**

- When and how were you first diagnosed, or suspect you have bipolar disorder?
- What was your response when you received your diagnosis?
- What does your diagnosis mean to you?
- Did you initially endorse the diagnosis? Why/why not?
- How does your diagnosis influence your sense of self?
- How does your diagnosis impact your daily life?
- How often do you share with others that you have a diagnosis of bipolar disorder? Why would or wouldn't you share that with someone?
- How do people normally respond when you share your diagnosis?
- What kinds of treatment are you on, or have been on? Can you share your thoughts?
- Have you ever been hospitalized for your bipolar disorder? Can you share your thoughts?

1. **Perceptions of bipolar from others**

- How would your family and friends describe bipolar disorder? Do you consider this accurate? Why/why not?
- How do you think the public would describe bipolar disorder? Do you consider this accurate? Why/why not?
- Can you describe an instance where you felt understood or seen as it pertains to your condition?
- Can you describe an instance where you did not feel understood or seen in your condition?
- Are there any misconceptions or stereotypes surrounding the bipolar disorder you’d like to express? Are there particular misconceptions about the (hypo)manic stages versus depressive stages?

1. **Ineffable mood states**

- How do you know when/ if you are in either mood state?
- Do you have any symptoms or behaviors that signal to you that you may be entering one or another?
- Do you have any symptoms or behaviors that signal to you that you may be exiting one or another?
- Do you feel you can articulate to others what it feels like to be in those “in-between” states?
- What do you do when you are questioning or feeling the onset of one stage versus another?

1. **Romanticization/mania**

- How do you think people understand mania specifically? Is it an accurate depiction? Why or why not?
- How do you feel when you are (hypo)manic? Can you provide examples of your experiences?
- Are there any positive aspects of your (hypo)mania? Can you give me an example?
- Do you feel others can notice shifts in your mood during manic or hypomanic phases? Can you provide an example?

1. **Depressive Phase**

- How would you describe your experiences during depressive phases? Can you share what it feels like and how it affects your daily life?
- Are there any misconceptions or stereotypes surrounding the depressive aspect of bipolar disorder that you'd like to address?
- Have you encountered any challenges or difficulties in explaining or getting support for your depressive episodes?
- Are there any positive aspects of your depressive states/episodes? Can you give me an example?

1. Is there anything else you’d like to add, or to ask me? Have we missed anything important?
